# Cell deformability of peripheral blood mononuclear cells is reduced in individuals with major depressive disorder

**DOI:** 10.1038/s41398-026-04225-w

**Published:** 2026-07-21

**Authors:** Lisa Kwapich, Tobias Neckernuss, Daniel Geiger, Jonas Pfeil, Kathrin Woike, Eun-Jin Sim, Markus Kiefer, Carlos Schönfeldt-Lecuona, Alexander Behnke, Marion Schneider, Othmar Marti, Iris-Tatjana Kolassa, Alexander Karabatsiakis

**Affiliations:** 1 Institute of Experimental Physics, Ulm University, 89081 Ulm, Germany; 2 Clinic for Psychiatry and Psychotherapy III, Ulm University Hospital, 89075 Ulm, Germany; 3 Department of Clinical & Biological Psychology, Institute of Psychology & Education, Ulm University, 89081 Ulm, Germany; 4 Department of Experimental Anesthesiology, Clinic for Anaesthesiology and Intensive Care Medicine, Ulm University Hospital, 89081 Ulm, Germany; 5 German Center for Mental Health (DZPG), partner site Mannheim-Heidelberg-Ulm, Ulm, Germany; 6 German Center for Child and Adolescent Health (DZKJ), partner site Ulm, Ulm, Germany

**Keywords:** Depression, Physiology

## Abstract

Major depressive disorder (MDD) is a complex mental disorder with a pathophysiology that remains only partly understood and involves, among other factors, alterations in immune system function. One of the main risk factors for mental disorders, in particular trauma-spectrum disorders like depression and complex PTSD, is childhood maltreatment (CM). However, their impact on the biophysical properties of immune cells remains unexplored. This study investigated PBMC deformability in 26 individuals diagnosed with MDD and 28 healthy controls. CM was assessed using the Childhood Trauma Questionnaire (CTQ). A microfluidic deformability assay was employed to assess immune cell deformability, with cell viability controlled in PBMC samples before measurement using trypan blue staining.

PBMCs from individuals with MDD exhibited significantly reduced deformability compared to those from healthy controls, indicating increased cellular stiffness. CM was not significantly associated with PBMC deformability, suggesting that the MDD phenotype itself, with its multiple potential etiological causes, plays a more crucial role in cell stiffness than any single risk factor. These findings suggest that immune cell stiffness is elevated in MDD when assessed in isolated PBMCs, contrasting with previous studies that reported increased deformability measured in whole blood.

Based on our working hypothesis, we interpret our results as indicative of damage to intracellular structures, including the cytoskeleton and cell membrane, likely due to increased exposure to free radicals from chronic or excessive stress. This may compromise membrane integrity and alter fatty acid composition. Functionally, these findings could enhance the biophysical understanding of impaired wound healing and other immunological dysfunctions commonly observed in MDD.

## Introduction

Major depressive disorder (MDD) is a significant global health issue, affecting approximately 280 million individuals worldwide [1]. With a lifetime prevalence of about 13% [2], MDD is a leading cause of disability. Childhood maltreatment (CM), which encompasses experiences of abuse and neglect before the age of 18, is a well-established risk factor for mental disorders, including MDD [3]. Women are generally more vulnerable to CM and are twice as likely as men to develop the disorder [2, 4]. In addition to persistent sadness and anhedonia, MDD is characterized by cognitive impairments, disturbances in sleep and appetite, reduced energy, and psychomotor changes. MDD also commonly co-occurs with anxiety disorders and other somatic and psychiatric comorbidities [5]. Despite extensive research and strong efforts to provide a comprehensive explanatory model for MDD, its precise biological underpinnings remain elusive.

The etiology of MDD involves a complex interplay of psychosocial, genetic, and physiological factors. A key component of these physiological alterations is the dysregulation of the hypothalamic-pituitary-adrenal (HPA) axis, the body’s central stress-response system [6, 7]. Studies have reported elevated levels of HPA axis-related signaling molecules, including corticotropin-releasing hormone (CRH), adrenocorticotropic hormone (ACTH), and cortisol, in individuals with MDD, suggesting impaired feedback regulation [8–11]. These alterations reflect broader changes in the synthesis, release, and regulation of stress-related signaling molecules, including hormones and other biomolecules influenced by chronic stress. However, these findings remain inconsistent, indicating that HPA axis abnormalities are not universal among patients and may depend on symptom severity, illness course, and chronicity [11].

The monoamine hypothesis posits that deficiencies in neurotransmitters such as serotonin, dopamine, and norepinephrine contribute to MDD [12, 13]. While widely accepted, this hypothesis has been challenged by conflicting evidence from neuroimaging and pharmacological studies [14]. Furthermore, reduced hippocampal volume and impaired neurogenesis have been observed in patients with MDD [15–18], potentially contributing to the cognitive and emotional deficits associated with depression. It still remains unclear whether reduced neurogenesis and neurotrophic factor levels are a cause or consequence of MDD-related abnormalities. In addition, the suspected “classical” neurotransmitters, including serotonin, are also involved in inflammatory processes like thermoregulation and immunomodulatory signaling [19]. In line with this, pro-inflammatory cytokines such as interleukin (IL)-1, IL-6, and tumor necrosis factor (TNF)-α are often elevated in MDD [20–25]. Taken together, alterations in neurotransmission, neuroplasticity, and inflammatory signaling suggest a more systemic disturbance of cellular stress responses in MDD. These processes are closely linked to oxidative stress pathways and cytoskeletal regulation, which may influence the mechanical properties of immune cells.

Findings of elevated markers of inflammation, oxidative stress and alterations in mitochondrial function have suggested impaired energy metabolism and cellular distress in MDD pathology [20–30]. Inflammation and oxidative stress are known influencing factors on cytoskeleton dynamics and membrane fluidity [31, 32]. For example, pro-inflammatory cytokines, including IL-6 and TNF-α, can impact cytoskeletal dynamics by promoting actin polymerization and altering microtubule stability, leading to increased cell stiffness. Additionally, oxidative stress has been shown to modify cytoskeletal proteins and membrane lipids, further impairing cell flexibility [33, 34]. In addition to these biological alterations, recent evidence highlights changes in the biophysical properties of biological systems as a potential contributor to the pathophysiology of MDD [35]. Deformability cytometry, a novel approach to studying MDD, has shown characteristic changes when examining whole blood [35]. The mechanical properties of cells, such as stiffness and deformability, are influenced by underlying cytoskeletal and membrane changes [36–38], which are themselves affected by oxidative stress (free radical production), lipid composition, and cytoskeletal dynamics – factors that are all altered in MDD [39–42]. Notably, leukocytes exhibit changes in deformability during immune activation [43], suggesting that their mechanical characteristics are linked to systemic changes associated with MDD [35, 44, 45]. Therefore, integrating biophysical properties, including immune cell mechanics, into research on MDD may help bridge gaps between disciplines and enhance our understanding of the disorder.

This study explored the mechanical phenotyping of peripheral blood mononuclear cells (PBMCs) collected from individuals with and without MDD, testing the hypothesis that patients with MDD exhibit increased cellular stiffness, which correlates with depression severity and trauma load from adverse childhood experiences.

## Materials and methods

### Recruitment and clinical characterization of study participants

Individuals with MDD were recruited from the Clinic of Psychiatry and Psychotherapy III at Ulm University, while healthy controls were recruited through public announcements and newspaper ads. Written informed consent was obtained from all participants. The study was approved by the ethics review board of Ulm University (number 169/12) and all methods were performed in accordance with the relevant guidelines and regulations. Data and samples from the cohort included sociodemographic and clinical data (Table 1) as well as blood samples from 26 patients with MDD and 28 healthy control participants. The patient group comprised individuals diagnosed with MDD based on DSM-IV-TR [46] criteria. All participants underwent interviews conducted by trained psychologists using standardized diagnostic tools to assess mental health conditions, including depression, anxiety disorders, and traumatic life events. Key inventories used included the short version of the Diagnostic Interview for Mental Disorders [47] (Mini-DIPS) for identifying mental health conditions and comorbidities, and the Beck Depression Inventory II [48] (BDI-II, self-report) to evaluate acute depressive symptom severity. Healthy controls also completed the Mini-DIPS as well as the BDI-II to screen individuals for mental disorders in general and depressive symptoms in particular. Traumatic events in childhood were assessed with the Childhood Trauma Questionnaire [49] (CTQ, self-report). Lifetime trauma and symptoms of a posttraumatic stress disorder were evaluated using the Essen Trauma Inventory [50] (ETI, self-report). Additional demographic and health-related variables, including education, body mass index (BMI), physical activity, physical illnesses, smoking status (current and lifetime), and alcohol consumption, were also collected. Table 1 summarizes the available information by variable. Individuals in the patient group met the criteria of a main diagnosis of MDD without comorbid mental disorders (e.g., addiction disorders, posttraumatic stress disorder or anxiety disorders) or somatic illnesses (e.g., cardiovascular diseases or diabetes mellitus). For a detailed description of clinical and medical characteristics of the study cohort, we refer to previously reported findings [51, 52]. Exclusion criteria for all participants included acute medical conditions, neurological disorders (e.g., seizures, Tourette syndrome, or somatic brain disorders), hyperthyroidism, pheochromocytoma, uncorrected vision or hearing impairments, pregnancy, alcohol or drug abuse, psychotic episodes, cluster A or B personality disorders, manic episodes, and inability to provide informed consent. Healthy control participants were free from lifetime mental disorders.

**Table 1:** Sociodemographic and clinical characteristics of patients with MDD and healthy control participants.

	Controls (n = 28)^a^	MDD (n = 26)^a^	Statistics^b^	*p*^c^
Sex *male* *female*	4 (14.3) 24 (85.7)	5 (19.2) 21 (80.8)	χ²(1) = 0.01	0.724
Age (years)	40.0 ± 10.6	35.9 ± 13.1	*t*(48.24) = 1.27	0.212
School education (years)^d^	9.9 ± 1.2	10.6 ± 1.5	W = 260	0.090
BMI (kg/m^2^)	25.7 ± 4.2	25.5 ± 5.4	*t*(46.95) = 0.09	0.927
Regular sports^d^	20 (71.4)	18 (69.2)	χ²(1) < 0.01	>0.999
Smoking *current* *lifetime*^*d*^	6 (21.4) 9 (32.1)	9 (34.6) 12 (46.2)	χ²(1) = 0.60 χ²(1) = 0.85	0.366 0.356
Occasional alcohol consumption	0 (0)	9 (34.6)	χ²(1) = 9.27	**<0.001**
Medication^e^				
*Antidepressants*	0 (0)	23 (88.5)	χ²(1) = 39.60	<0**.001**
*Benzodiazepines*	0 (0)	9 (34.6)	χ²(1) = 9.27	<0**.001**
*Antipsychotics*	0 (0)	5 (19.2)	χ²(1) = 3.87	**0.021**
Depressive symptoms (BDI-II sum score)	2.4 ± 2.2	32.2 ± 9.7	*t*(27.45) = −15.38	**<0.001**
Childhood maltreatment				
CTQ sum score^d^	36.9 ± 11.8	44.0 ± 11.7	*t*(43.25) = −2.08	0.**043**
CTQ classification^f^	9 (32.1)	13 (50)	χ²(1) = 2.29	0.130
Traumatic event exposure (ETI sum score)^d^	3.0 ± 2.1	3.0 ± 1.9	*t*(42.82) = 0.06	0.950
Storage time of samples at −80 °C (years)	2.8 ± 0.5	2.2 ± 0.6	*W* = 584	**<0.001**
PBMC viability after thawing (%)^g^	52.7 ± 10.1	59.0 ± 13.2	*t*(46.94) = −1.97	0.055
PBMC viability after MACS (%)^g^	81.9 ± 5.3	82.6 ± 7.2	*t*(45.97) = −0.40	0.692
PBMC viability after DC (%)^g^	32.4 ± 7.6	33.5 ± 11.1	*t*(38.19) = 0.40	0.689

*BDI-II*, Beck Depression Inventory II; *BMI*, Body mass index; *CTQ*, Childhood Trauma Questionnaire; *DC*, Deformability cytometry; *ETI*, Essen Trauma Inventory; *MACS*, magnetic-activated cell sorting; *MDD*, Major depressive disorder.

^a^Data represent n (%) for categorical variables and mean ± standard deviation for continuous variables.

^b^Two-tailed Welch’s *t*-test (*t)*, Mann-Whitney *U* test (W), or Chi-squared test (*χ2*).

^c^Bold *p*-values indicate significance at an alpha level of 0.05.

^d^Not available data on school education from one MDD patient, on physical activity from one MDD patient, on lifetime smoking from one control participant and two MDD patients, on CTQ sum score from one control participant and five MDD patients, on ETI sum score for one control participant and six MDD patients.

^e^Benzodiazepines and antipsychotics were adjunctive treatment to antidepressants.

^f^Binary classification for childhood maltreatment exposure according to the moderate CTQ classification cutoff. Data not available for one control participant and four MDD patients.

^g^PBMC viability after thawing as well as after MACS was assessed by trypan blue staining, while PBMC viability after DC was determined by propidium iodide staining using flow cytometry.

### Sample preparation

Peripheral blood samples were collected via venipuncture into EDTA-buffered collection tubes (Sarstedt). Participants were not required to fast prior to blood collection. PBMCs were isolated using Ficoll-Hypaque density gradient centrifugation following the manufacturer’s protocol (Leucosep, Greiner Bio-One). The collected buffy coat was washed and resuspended in a standard cryoprotective freezing medium (1:10 dilution of dimethyl sulfoxide (DMSO, Sigma) in heat-inactivated fetal bovine serum (Sigma)). PBMC aliquots were stored at −80 °C until further analysis. To ensure objectivity, samples were coded, and laboratory measurements were performed blinded to minimize experimenter bias.

On the day of analysis, cryopreserved PBMC samples were rapidly thawed at 37 °C and washed by transferring the suspension into a 15 ml Falcon tube containing 10 ml of Dulbecco’s phosphate-buffered saline (DPBS, Gibco, 4 °C), followed by centrifugation at 232 g at 4 °C for 10 min. The supernatant was discarded, and PBMCs were resuspended in 10 ml of DPBS (Gibco, 4 °C). To assess cell viability, a 10 µl aliquot was stained with 0.4% trypan blue solution (Sigma). Live and dead cells were counted using a Neubauer counting chamber under a bright-field microscope (Axiovert 40 CFL, Zeiss). The washing step was repeated to remove any DMSO and cell debris. Finally, PBMCs were resuspended in 2 ml of DPBS (4 °C) for subsequent preanalytical processing.

### Magnetic-activated cell sorting

During the establishment of the microfluidic deformability assay in the laboratory, clogging of the microfluidic channel was observed. This issue was traced to the activation of CD15^+^ neutrophil granulocytes, which were detected in the isolated cell fraction via flow cytometry. To obtain a PBMC fraction with minimal contamination, CD15^+^ cells were depleted by magnetic-activated cell sorting (MACS) using CD15 MicroBeads (Miltenyi Biotec). Additionally, dead cells were removed through this MACS-based depletion.

MACS separation was performed according to the manufacturer’s protocol for up to 10⁷ cells. The buffer used was prepared by dissolving 0.5% bovine serum albumin (BSA, Sigma) in DPBS (Gibco), further referred to as MACS buffer. Although the manufacturer recommends adding EDTA, it was omitted as it inhibits *DNase I*, which was used post-MACS separation to digest any available extracellular DNA from neutrophil stimulation. The cell suspension was centrifuged at 300 g at 4 °C for 10 min, and the supernatant was completely aspirated. The cell pellet was resuspended in 80 µl of MACS buffer, and 20 µl of CD15 MicroBead solution (Miltenyi Biotec) was added. The suspension was thoroughly mixed and incubated at 4 °C for 15 min. After incubation, cells were washed with 2 ml of MACS buffer, centrifuged at 300 g at 4 °C for 10 min, and the supernatant was aspirated. The cells were then resuspended in 500 µl of MACS buffer. Before magnetic separation, the cells were passed through a 35 µm cell strainer (BD Biosciences) to achieve a single-cell suspension and remove clumps that might clog the column. A MACS column (type MS) was placed in the magnetic field of an OctoMACS separator (Miltenyi Biotec) and prepared by rinsing with 500 µl of MACS buffer. The cell suspension was applied to the column once the reservoir was empty. The unlabeled CD15^-^ cells that passed through the column were collected, and the column was washed three times with 500 µl of MACS buffer. The effluents from the washing steps were pooled with the unlabeled cell fraction.

### *DNase I* treatment

To further prevent cell clumping, *DNase I* (Roche) was added to the purified cell suspension, as it has been demonstrated that treating PBMCs with DNase after thawing effectively prevents aggregate formation without cytotoxic effects at concentrations up to 1 mg/ml. *DNase I* was added at a working concentration of 0.1 mg/ml and mixed briefly. The cells were incubated at 37 °C for 10 min to achieve optimal enzymatic activity. Following incubation, cell viability was reassessed using trypan blue staining for adjusting cell concentration. The cells were washed by centrifugation at 300 g at 4 °C for 10 min, and the supernatant was removed. The cell pellet was resuspended in DPBS (Gibco, 4 °C) at a concentration of 0.1 ×10⁶ to 5.0 ×10⁶ cells/ml, depending on the total cell count.

### Device fabrication

Negative photomasks were designed using KLayout [53] software and fabricated on soda-lime glass via electron beam lithography. Silica masters were created using soft photolithography techniques. Specifically, a Ti-primer adhesion reagent (MicroChemicals) was applied to a 2” silicon wafer (MicroChemicals), spun at 500 rpm for 10 s and then at 5000 rpm for 60 s (Convac 1001). The wafer was baked at 120 °C for 2 min. A negative photoresist (SU-8 10, Microchem) was then spun onto the wafer at 500 rpm for 10 s and at 3000 rpm for 60 s (Opti spin ST23-1). The coated wafer was soft-baked at 65 °C for 2 min, followed by 95 °C for 5 min. For cleaning, the mask was subjected to ultrasonic cleaning in an ultrasonic bath, first in acetone and then in isopropanol for 4 min, respectively. The wafer was exposed to UV light on a mask aligner (MJB3, Suss MicroTec), followed by a post-exposure bake at 65 °C for 2 min and 95 °C for 5 min. The exposed photoresist was developed using SU-8 Developer (mr DEV 600, Microresist Technology) for approximately 2 min, ensuring that a rinse with isopropyl alcohol produced no white film. The silicon wafer was placed on the heating plate at 95 °C for 5 min to harden, followed by 175 °C for 30 min. The mold was secured to the lower plate of a petri dish with the features facing upward, and a layer of Sylgard 184 Silicone Elastomer (Dow) containing polydimethylsiloxane (PDMS) with a 10:1 weight/weight ratio of base to curing agent was poured over it. The mold was placed in a vacuum chamber, which was evacuated for 30 min to remove air from the polymer. It was then cured in an oven at 65 °C for at least 2 h. The resulting microfluidic chip was cut from the mold and stored on a glass slide. For the microfluidic deformability assay, the microfluidic chip and a cleaned glass slide were activated in a plasma cleaner (Femto version A, Diener Electronic). After air plasma treatment, the activated surfaces of the PDMS and glass were brought into contact, forming permanent covalent bonds. To ensure consistent surface properties, microfluidic deformability assay experiments were conducted 24 h after plasma treatment.

### Experimental setup

The microfluidic chip was mounted on the XY stage of an inverted bright-field microscope (Axio Observer D1, Zeiss). Microfluidic deformability assay experiments required a minimum absolute sample volume of 350 μl. Cell suspensions were transferred into a 1 ml syringe. The syringe was connected to the input port of a modified glass slide using polymer tubing, which had been cleaned with 100% isopropanol followed by Milli-Q water. The input port on the glass slide directed the cell suspension to the inlet of the microfluidic chip, where the flow channel commenced. The channel featured a 250 µm-long constriction with a cross-sectional area of 10 ×15 µm (width x height) and terminated at the outlet of the chip. The outlet was connected to the output port of the modified glass slide, which was in turn linked via cleaned polymer tubing to a 1.5 ml tube for collecting the flow-through. A high-precision syringe pump (Pump 11 Pico Plus Elite, Harvard Apparatus) was employed to drive the cell suspension through the channel at a constant flow rate of 10 µl/min. Before measurements began, the flow was stabilized for 1.5 min. All measurements were carried out at room temperature.

### Data analysis

A custom-developed algorithm in Matlab v2018a [54] was employed for image analysis, data collection, and postprocessing. The algorithm was designed to detect the presence of cells, to identify their contours, and to quantify various parameters. The analysis generates a dataset of parameters including intensity, length (maximum horizontal diameter), width (maximum vertical diameter), area (cell size), circumference, and velocity. Deformability is calculated as the ratio of length to width, with a perfect circle having a deformation value of exactly one. For each cell, parameters are extracted from all frames where the entire cell is visible within the field of view, allowing for the computation of average values to minimize noise. A Fischer pattern was used for pixel-to-micrometer conversion. To exclude cell debris from the analysis, several filters were applied. Only objects meeting the following criteria were identified as cells: length < 20 µm, width < 10 µm, deformability between 0.5 and 1.5, area > 12 µm², circumference > 2 µm, velocity between 0.5 ×10⁵ and 2.0 ×10⁵ µm/s, and pixelation factor ≤ 1. For each participant, between approximately 200 and 27 000 individual cells were recorded, depending on sample quality and cell yield. Of these, around 22% met the predefined filtering criteria and were included in the analysis. To derive one representative value per individual, the distribution of single-cell measurements (e.g., deformability, size) was summarized using the median rather than the mean, as these distributions were typically skewed and the mean would have been disproportionately influenced by extreme values.

### Flow cytometry

Flow cytometry was conducted for immunological fingerprinting of PBMCs using a FACSCalibur flow cytometer (BD Biosciences) following microfluidic deformability analyses. Flow cytometry was conducted using a panel of monoclonal antibodies targeting CD45 (1:100), CD14 (1:100), TCRα/β (1:100), CD3 (1:100), CD4 (1:100), CD8 (1:100), CD16 (1:100), CD56 (1:100), CD5 (1:100), CD19 (1:100), CD163 (1:100), and CD80 (1:22), along with corresponding isotype controls (Miltenyi Biotec). Cell surface staining was performed according to the manufacturer’s protocol for up to 10⁶ cells. The buffer used for staining was prepared by dissolving 0.5% BSA (Sigma) in DPBS (Gibco), further referred to as FACS buffer. Cells were centrifuged at 300 g at 4 °C for 10 min. The supernatant was completely aspirated, and the cell pellet was resuspended in 1 ml of FACS buffer. A volume of 100 µl of the resuspended cells was transferred into each of nine flow cytometry tubes. The appropriate antibody combinations, consisting of one FITC-labeled antibody and one PE-labeled antibody, were added as detailed in Table 2. To additionally assess cell viability, the cell suspension in the ninth tube was stained with propidium iodide (5 µg/ml, Sigma). The tubes were mixed thoroughly and incubated at 2–8 °C in the dark for 10 min. Following incubation, the cells were washed by adding 2 ml of FACS buffer and centrifuged at 300 g at 4 °C for 10 min. The supernatant was decanted, leaving approximately 200 µl for flow cytometric analysis.

**Table 2:** Antibody combinations for flow cytometry.

Tube	FITC ab	Clone	Isotype		PE ab	Clone	Isotype
#1	Isotype control	REA293	rh-IgG1	+	Isotype control	REA293	rh-IgG1
#2	CD45	REA747	rh-IgG1	+	CD14	REA599	rh-IgG1
#3	TCRα/β	REA652	rh-IgG1	+	CD3	REA613	rh-IgG1
#4	CD4	REA623	rh-IgG1	+	CD8	REA734	rh-IgG1
#5	CD16	REA423	rh-IgG1	+	CD56	REA196	rh-IgG1
#6	CD5	REA782	rh-IgG1	+	CD19	REA675	rh-IgG1
#7	CD163	REA812	rh-IgG1	+	CD80	REA661	rh-IgG1
#8	Negative control			+	Negative control		

### Statistics

Statistical analyses were conducted using R version 4.3.3 [55]. Comparisons between study groups were performed using *χ*^2^ tests for categorical variables and either Welch’s *t*-tests or Mann-Whitney *U* tests for continuous variables, as appropriate. Residual distributions and variance homogeneity were evaluated using diagnostic plots. Bivariate associations were examined using Spearman correlations (*r*_S_). Two-way ANOVAs (2 × 2) were used to examine the combined influence of diagnostic group and childhood maltreatment (CM) on the biological readouts. Tests with *p*-values < 0.05 (two-tailed) were considered statistically significant.

## Results

### Reduced PBMC deformability in patients with MDD

**Fig. 1:**
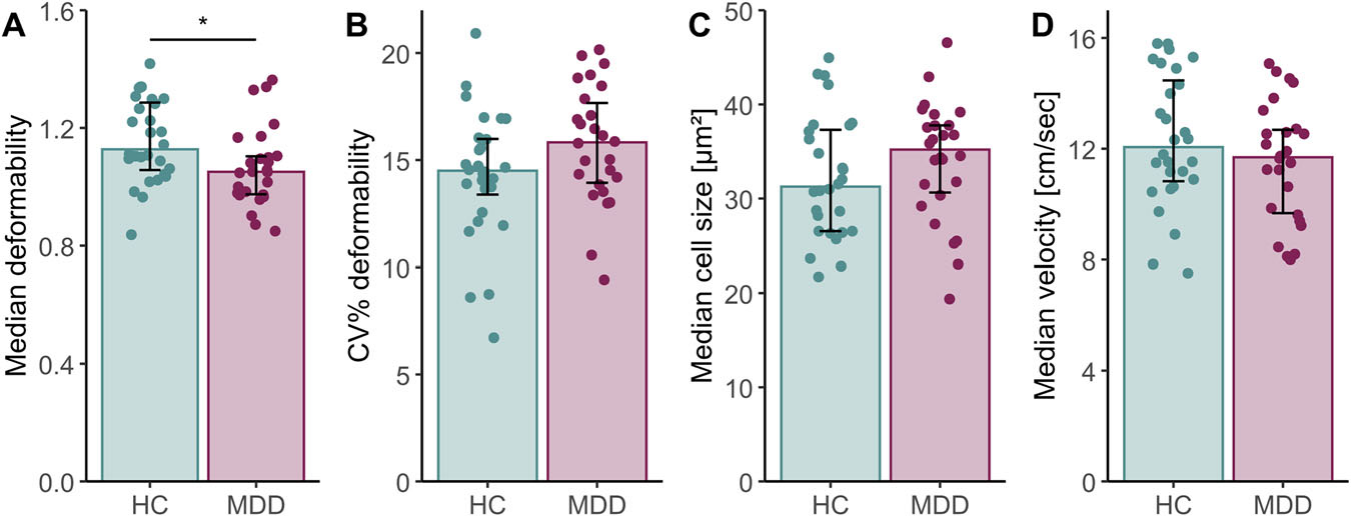
PBMC deformability, size, and velocity in patients with major depressive disorder and healthy controls. Comparison of patients with major depressive disorder (MDD: *n* = 26) and healthy controls (HC: *n* = 28) with respect to (**A**) median PBMC deformability, (**B**) intraindividual variability in PBMC deformability, expressed as the coefficient of variation (CV), (**C**) median PBMC size, and (**D**) median PBMC velocity. Dots represent individual participants. Bars and error bars indicate the median and interquartile range, respectively. Significant group differences are indicated *p < 0.050, two-tailed.

PBMCs from patients with MDD showed significantly lower deformability, *M(SD)* = 1.07 (0.13), compared to PBMCs from healthy control participants, *M(SD)* = 1.16 (0.14), *t*_Welch_(51.94) = 2.45, *p* = 0.018, *d*_Cohen_ = 0.67 (Fig. 1A). In line with this, greater depressive symptom severity on the BDI-II sum score was marginally linked to lower PBMC deformability (*r*_S_ = −0.23, *p* = 0.094). There was no significant difference in the intraindividual variability of cell deformability between the groups (coefficient of variation MDD vs. controls: *M(SD)* = 15.7% (2.8%) vs. 14.3% (3.0%), *t*_Welch_(51.98) = −1.78, *p* = 0.082, *d*_Cohen_ = −0.48, Fig. 1B). As cell deformation in the constriction of the flow channel is influenced not only by cell stiffness but also by cell size and velocity, median cell size and median velocity were also compared between study groups. No significant group differences were found for median cell size, MDD vs. controls: *M(SD)* = 34.08 µm² (6.31 µm²) vs. 32.25 µm² (6.50 µm²), *t*_Welch_(51.89) = −1.05, *p* = 0.297, *d*_Cohen_ = −0.29 (Fig. 1C), and median flow velocity, MDD vs. controls: *M(SD)* = 11.51 cm/s (21.58 cm/s) vs. 12.31 cm/s (23.79 cm/s), *t*_Welch_(51.97) = −1.30, *p* = 0.198, *d*_Cohen_ = 0.35 (Fig. 1D).

### Childhood maltreatment and PBMC deformability

CM causes enduring negative alterations in various biological systems, such as the stress and inflammatory system, which likely contribute to the increased lifetime vulnerability of individuals with a history of CM for subsequent mental health problems [56, 57]. However, on a bivariate level, there was no significant association between PBMC deformability and CM exposure on the CTQ sum score, neither in the entire cohort (*r*_S_ = −0.23, *p* = 0.121), nor in the MDD (*r*_S_ = 0.22, *p* = 0.350) and control group (*r*_S_ = −0.25, *p* = 0.217). We additionally explored whether CM exposure (defined by the moderate cutoff on the CTQ) would account for differences in cell deformability beyond the clinical diagnosis of MDD. However, a 2 × 2 ANOVA, *F*(3,45) = 3.64, *p* = 0.020, R² = 0.195, indicated no significant main effect of CM exposure, *F*(1,45) = 0.36, *p* = 0.553, η² = 0.007, and no significant interaction of diagnostic group × CM exposure, *F*(1,45) = 0.17, *p* = 0.680, η² = 0.003, beyond the significant difference between MDD patients and controls, *F*(1,45) = 8.53, *p* = 0.005, η² = 0.158. Likewise, there were no CM group or interactions effects on interindividual variability of PBMC deformability, the average cell size and velocity.

### Explorative and sensitivity analyses

Correlation analyses and tests for group differences indicated no significant associations of PBMC deformability with potential influences such as age (*r*_S_ = 0.01, *p* = 0.926), BMI (*r*_S_ = 0.02, *p* = 0.862), sex (*t*_Welch_(11.28) = −1.09, *p* = 0.301), current smoking (*t*_Welch_(25.39) = 0.98, *p* = 0.339), occasional alcohol consumption (*t*_Welch_(12.01) = 0.83, *p* = 0.425), and lifetime traumatic event exposure (ETI: *r*_S_ = 0.20, *p* = 0.186). PBMC deformability was lower among subjects receiving antidepressant medication than those without (*t*_Welch_(50.28) = 2.41, *p* = 0.020, *d*_Cohen_ = 0.66). However, because the vast majority of participants with MDD (88.5%) with only three exceptions were receiving antidepressant medication, the potential impact of the pharmacological treatment on cell deformability could not be disentangled from the cohort’s clinical diagnoses. Considering methodological confounders, we observed that PBMC samples that were stored for a longer time at −80 °C exhibited a higher average deformability (*r*_S_ = 0.64, *p* < 0.001) and a lower variability in the deformability of single cells within one sample (*r*_S_ = −0.57, *p* < 0.001).

## Discussion

### Increased PBMC stiffness in MDD

This study observed increased cell stiffness, i.e., significantly reduced deformability of PBMCs from individuals with MDD compared to those from healthy controls. The reduction in PBMC deformability was not accompanied by significant differences in cell size or velocity, indicating that the observed mechanical changes are primarily due to alterations in cellular stiffness rather than overall cell morphology or flow characteristics. Contrary to expectations, CM had no significant effect on PBMC deformability, despite previous research linking it to increased oxidative stress and lipid peroxidation [58]. This suggests underlying cytoskeletal and membrane alterations in immune cells [59, 60]. These findings indicate that PBMC deformability is more closely associated with MDD itself rather than childhood adversity, which is just one of many potential risk factors for MDD.

MDD has been associated with impaired tissue healing [61, 62], potentially linked to altered immune cell-cell interactions, reduced migration capacity, and changes in the induction or secretion of inflammatory mediators. At the cellular level, reduced PBMC deformability may indicate impaired immune cell adaptability, potentially driven by a pro-inflammatory environment and intracellular oxidative stress that in sum might alter cytoskeletal properties and membrane characteristics.

Moreover, immune-related mechanisms such as the status of activation of an immune cell could contribute to the observed decrease in PBMC deformability in MDD. Immune cells that proliferate and migrate into tissue require greater motility and often exhibit increased deformability. Proliferating immune cells, particularly those involved in tissue repair and inflammation resolution, need to efficiently migrate through endothelial barriers and tissue environments, necessitating greater mechanical flexibility.

A previous study reported higher deformability of blood cells when analyzed in blood droplets [35]. In contrast to our approach, whole blood contains a significant proportion of red blood cells, which have distinct biophysical characteristics that heavily influence the deformability profile of the sample. Isolated PBMCs, on the other hand, lack red blood cells. Therefore, it is crucial to differentiate the contributions of individual blood cell populations to the overall deformability signal. The contrasting findings emphasize the importance of examining deformability characteristics in isolated blood cell populations (e.g., red blood cells, lymphocytes, monocytes) to better understand and define the underlying nature of such a profile.

### Strengths, limitations, and methodological considerations

One strength of this study is the assessment of cell viability both before and after microfluidic deformability experiments to evaluate sample quality. Interestingly, an increased proportion of non-viable cells was observed in the PBMC samples following deformability analyses. This suggests that flow cytometry analyses should be performed on separate samples rather than on the same sample after deformability cytometry. However, due to the limited number of PBMCs available for this study, it was not feasible to process them for both approaches in parallel as well as measuring technical batch controls.

Another strength and at the same time limitation of the study is that we used cryopreserved PBMCs in contrast to fresh PBMCs or whole blood which limits the comparability of findings in comparison to previously reported results [35]. Using cryopreserved PBMCs provides greater flexibility in experiment planning and execution. However, both the cryopreservation process and storage time can affect cell viability, highlighting the importance of co-assessing cell viability as a crucial confounder. In our experiments, the percentage of dead cells was lower than 20% for both groups before deformability cytometry. Therefore, the samples and the experimental analyses were considered to be of sufficient quality. Future studies should aim to minimize storage duration variability or use fresh samples to confirm that deformability differences are not driven by technical or methodological factors.

Notably, we observed a statistical positive association between storage time in the freezer and median deformability. However, this finding contradicts our expectations, as an increase in cell deformability over time seems unlikely. Importantly, storage duration differed significantly between samples from individuals with MDD and healthy controls. Therefore, the observed effect of freezing time may be driven by underlying group differences rather than a direct impact of storage duration on cell deformability.

In addition, our deformability metric represents a single scalar parameter and therefore does not capture the full viscoelastic behavior of immune cells. Cellular mechanics depend not only on elastic deformation but also on time-dependent viscous properties, cytoskeletal remodeling rates, and intracellular fluid dynamics. A scalar deformability value cannot resolve these complex viscoelastic contributions. Future studies using complementary techniques, such as real-time deformability cytometry variants, microfluidic rheology, or atomic force microscopy, will be necessary to obtain a more complete mechanical phenotype.

Finally, a more comprehensive understanding of the potential contributions of lifestyle factors (e.g., BMI, smoking, and sleep) and other health-related variables to cell deformability measures will require larger cohorts with improved balancing for sex and antidepressant medication use. However, because antidepressant treatment is more common among patients with severe depression, depressive symptom severity and medication use are intrinsically confounded and cannot be independently evaluated. Future studies should compare the effects of different antidepressants and their dosages on cell deformability.

### Future studies

Future studies should additionally investigate oxidative stress markers, lipid membrane composition, and cytoskeletal regulators in PBMCs. Furthermore, combining mechanical phenotyping with transcriptomics and proteomics approaches may provide a more comprehensive understanding of the pathways linking immune cell mechanics to MDD pathophysiology. In addition, omics approaches applied to blood plasma, e.g., metabolomics and lipidomics, could help to reveal biochemical signatures indicative of cellular changes associated with cell deformability. In a first step, associations between changes in proinflammatory signaling and deformability could be addressed to get first insights into these interactions.

Previous studies used whole blood for deformability analyses [35]. This study was the first to assess deformability in total PBMCs of MDD patients. Future studies should examine cell subtypes (e.g., monocytes, NK cells, T-cell subsets) as shifts in immune cell composition may contribute to the observed differences in PBMC deformability. Our exploratory flow-cytometry findings indicated altered proportions of NK cells and M2-like monocytes in MDD, and both subsets were positively associated with PBMC deformability. This suggests that compositional shifts, rather than or in addition to intrinsic mechanical alterations, might influence the aggregate mechanical signature of PBMCs demanding immune profiling in future studies.

## Conclusion

This study demonstrated that PBMCs from patients with MDD exhibit increased cellular stiffness, suggesting that altered immune cell mechanics may be a feature of the disorder. While childhood maltreatment has been linked to immune dysregulation in prior research, no effect on PBMC deformability was observed, indicating that these mechanical changes are more closely associated with MDD itself. These findings contrast with previous studies reporting increased deformability in whole blood cells, highlighting that the mechanical properties of isolated immune cells may reflect different biological mechanisms. The results support the hypothesis that intracellular damage to structures such as the cytoskeleton and cell membrane may contribute to increased stiffness in immune cells of individuals with MDD, potentially due to chronic exposure to free radicals and oxidative stress. Functionally, increased immune cell stiffness could help explain impairments in wound healing and broader immune dysfunctions frequently observed in MDD. Additionally, methodological factors such as cryopreservation effects and the presence of non-viable cells in the sample should be carefully considered in future studies. Understanding the interplay between immune cell mechanics, inflammation, and depression may pave the way for new therapeutic strategies targeting immune dysfunction in MDD.

## Supplementary information

Supplemental Material

## Data Availability

The participants of this study did not give written consent for their data to be shared publicly. Therefore, due to the sensitive nature of the research, supporting data is not available.
