# Supplementary material for: Cell deformability of peripheral blood mononuclear cells is reduced in individuals with major depressive disorder
Source: Transl Psychiatry. 2026 Jul 21;16:375. doi: 10.1038/s41398-026-04225-w (PMC13392435; doi:10.1038/s41398-026-04225-w)
Supplement: Supplementary file 1 — Supplemental Material [file 41398_2026_4225_MOESM1_ESM.pdf]

Cell deformability of peripheral blood mononuclear cells is reduced in individuals with major depressive disorder

Authors:

Lisa Kwapich *[1], Tobias Neckernuss [1], Daniel Geiger [1], Jonas Pfeil [1], Kathrin Woike [2], Eun-Jin Sim [2], Markus Kiefer [2], Carlos Schönfeldt-Lecuona [2], Alexander Behnke [3], Marion Schneider [4], Othmar Marti [1], Iris-Tatjana Kolassa [3,5,6], and Alexander Karabatsiakis [3]

Author affiliations:

[1] Institute of Experimental Physics, Ulm University, 89081 Ulm, Germany.

[2] Clinic for Psychiatry and Psychotherapy III, Ulm University Hospital, 89075 Ulm, Germany.

[3] Department of Clinical & Biological Psychology, Institute of Psychology & Education, Ulm University, 89081 Ulm, Germany.

[4] Department of Experimental Anesthesiology, Clinic for Anaesthesiology and Intensive Care Medicine, Ulm University Hospital, 89081 Ulm, Germany

[5] German Center for Mental Health (DZPG), partner site Mannheim-Heidelberg-Ulm, Ulm, Germany

[6] German Center for Child and Adolescent Health (DZKJ), partner site Ulm, Ulm, Germany

* Corresponding author:

Lisa Kwapich, Institute of Experimental Physics, Ulm University, 89081 Ulm, Germany, E-mail: lisa.kwapich@uni-ulm.de.

Supplementary results

Altered immune cell composition of PBMCs from patients with MDD

Different subpopulations of PBMCs exhibit different cytoskeletal features to perform their physiological purposes (e.g., migration to tissue). To investigate whether changes in PBMC composition, as previously reported in MDD[62], might account for the difference in PBMC deformability, the diagnostic groups were compared for compositional changes in PBMCs and the correlations between the PBMC subpopulation proportions and PBMC deformability were analyzed (**Supplementary Table 1**, **Supplementary Figure 1**). Overall, a lower relative abundance of natural killer (NK) cells ($CD16^+/CD56^+$, $d_{Cohen} = 0.96$, $p = .002$) and M2-like monocytes ($CD163^+/CD80^-$, $d_{Cohen} = 0.96$, $p = .034$) in PBMCs of patients with MDD was observed as compared to those of healthy control participants. This compositional difference might partially explain the lower deformability of mixed PBMCs in MDD, as a higher proportion of these cell types accounted for a higher average deformability in the overall PBMC population (M2-like monocytes: $r_S = 0.35$, $p = .010$; NK cells: $r_S = 0.24$, $p = .080$), which suits these cell types' physiological function of migrating into tissue.

Figure legends

Supplementary Figure 1. (A) Percentage fraction of monocytes ($CD45^{+}/CD14^{+}$), M1-like monocytes ($CD80^{+}/CD163^{-}$), M2-like monocytes ($CD163^{+}/CD80^{-}$), B cells ($CD19^{+}/CD5^{-}$), Tαβ cells ($TCR\alpha\beta^{+}/CD3^{+}$), T helper cells ($CD4^{+}/CD8^{-}$), cytotoxic T cells ($CD8^{+}/CD4^{-}$), and natural killer cells ($CD16^{+}/CD56^{+}$) in PBMCs of healthy control participants (HC: n = 28) and patients with MDD (MDD: n = 26). The percentage fraction refers to the total amount of gated PBMC populations assessed by flow cytometry after deformability cytometry. Bars and error bars indicate the median and interquartile range of variable distributions in groups. Significant group differences are indicated * $p < .050$, ** $p < .010$, two-tailed. Association of the relative proportion of (B) M1-like monocytes, (C) M2-like monocytes, (D) cytotoxic T cells, and (E) natural killer cells among total PBMCs with the median deformability of total PBMCs.

Supplementary tables

Supplementary Table 1. PBMC subset composition of patients with MDD and healthy control participants

	**Controls (n = 28)**[a]	**MDD (n = 26)**[a]	**Statistics**[b]	$\boldsymbol{p}$[c]	$\boldsymbol{r_S}$[d] **(*p*)**
**Monocytes** (CD45$^+$/CD14$^+$) (%)	23.8 ± 6.3	19.9 ± 9.1	$t(44.36) = 1.85$	.071	.09 (.509)
M1-like monocytes (CD80$^+$/CD163$^-$) (%)	0.8 ± 1.2	0.3 ± 0.5	$W = 404.0$	.494	**-.31*** (.021)
M2-like monocytes (CD163$^+$/CD80$^-$) (%)	27.9 ± 7.9	23.4 ± 7.4	$t(51.99) = 2.17$	**.034***	**.35*** (.010)
**Lymphocytes**					
B cells (CD19$^+$/CD5$^-$) (%)	14.1 ± 6.6	17.0 ± 7.6	$t(49.69) = -1.47$	.147	-.06 (.662)
Tαβ cells (TCRαβ$^+$/CD3$^+$) (%)	38.0 ± 10.9	40.3 ± 14.0	$t(47.38) = -0.67$	.504	-.17 (.206)
T helper cells (CD4$^+$/CD8$^-$) (%)	33.2 ± 6.5	33.2 ± 10.5	$t(41.40) = 0.01$	.998	.01 (.966)
Cytotoxic T cells (CD8$^+$/CD4$^-$) (%)	12.1 ± 8.8	12.9 ± 7.4	$t(51.53) = -0.35$	.727	**-.32*** (.014)
Natural killer cells (CD16$^+$/CD56$^+$) (%)	11.1 ± 5.5	6.9 ± 3.7	$t(47.79) = 3.33$	**.002****	.24 (.080)

[a] Data represent mean ± standard deviation. The percentage fraction refers to the total amount of gated PBMC populations assessed by flow cytometry.
[b] Two-tailed Welch's t-test (*t)* or Mann-Whitney *U* test (*W*).
[c] Bold *p*-values indicate significance on a two-tailed alpha level of 0.05.
[d] Spearman correlation with PBMC median deformability.

Supplementary figures

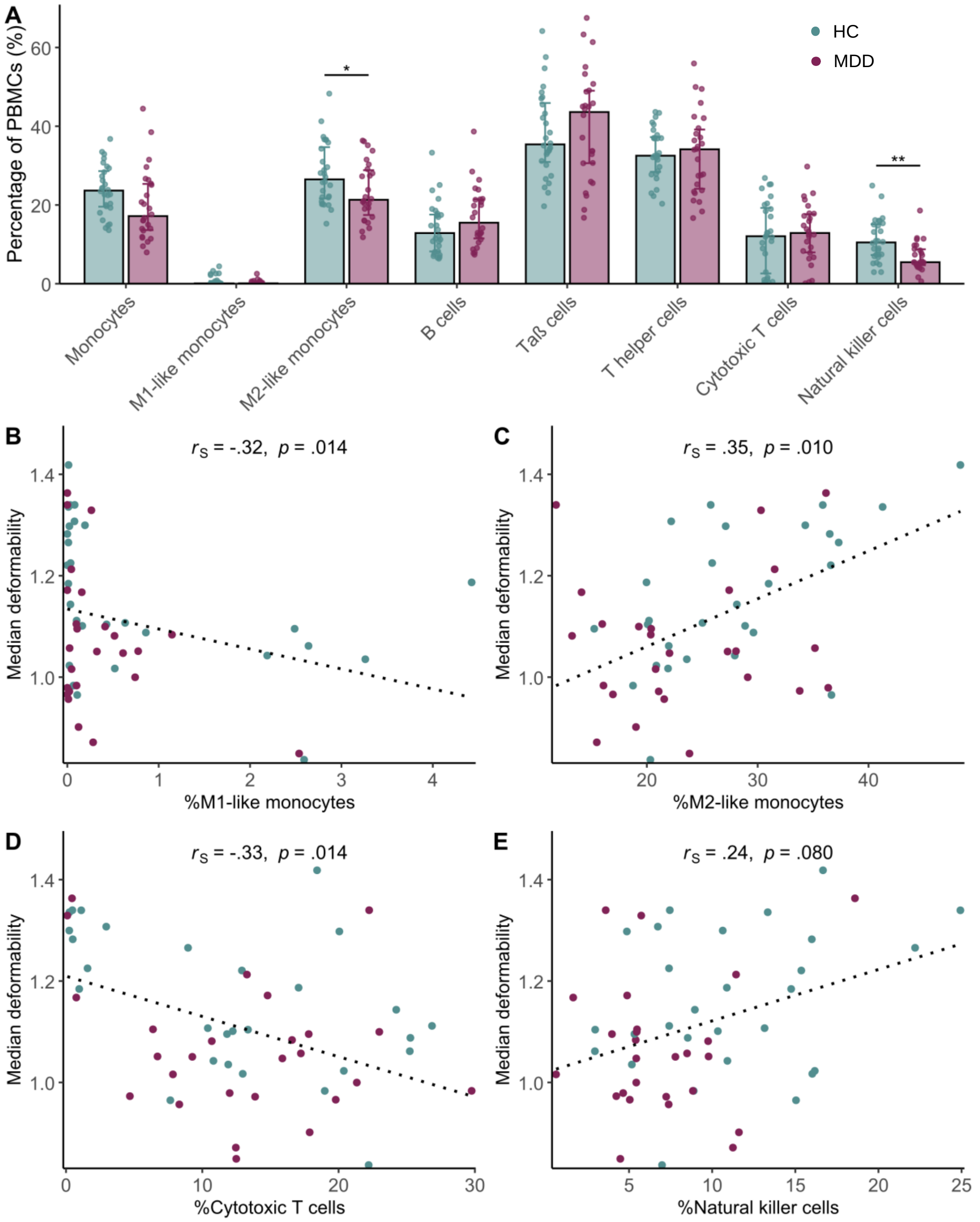

Supplementary Figure 1
